# E-Cadherin Is Required for Centrosome and Spindle Orientation in *Drosophila* Male Germline Stem Cells

**DOI:** 10.1371/journal.pone.0012473

**Published:** 2010-08-31

**Authors:** Mayu Inaba, Hebao Yuan, Viktoria Salzmann, Margaret T. Fuller, Yukiko M. Yamashita

**Affiliations:** 1 Center for Stem Cell Biology, Life Sciences Institute, University of Michigan, Ann Arbor, Michigan, United States of America; 2 Department of Cell and Developmental Biology, School of Medicine, University of Michigan, Ann Arbor, Michigan, United States of America; 3 Cellular and Molecular Biology Program, University of Michigan, Ann Arbor, Michigan, United States of America; 4 Departments of Developmental Biology and Genetics, Stanford University School of Medicine, Stanford, California, United States of America; New Mexico State University, United States of America

## Abstract

Many adult stem cells reside in a special microenvironment known as the niche, where they receive essential signals that specify stem cell identity. Cell-cell adhesion mediated by cadherin and integrin plays a crucial role in maintaining stem cells within the niche. In *Drosophila melanogaster*, male germline stem cells (GSCs) are attached to niche component cells (*i.e.*, the hub) via adherens junctions. The GSC centrosomes and spindle are oriented toward the hub-GSC junction, where E-cadherin-based adherens junctions are highly concentrated. For this reason, adherens junctions are thought to provide a polarity cue for GSCs to enable proper orientation of centrosomes and spindles, a critical step toward asymmetric stem cell division. However, understanding the role of E-cadherin in GSC polarity has been challenging, since GSCs carrying E-cadherin mutations are not maintained in the niche. Here, we tested whether E-cadherin is required for GSC polarity by expressing a dominant-negative form of E-cadherin. We found that E-cadherin is indeed required for polarizing GSCs toward the hub cells, an effect that may be mediated by Apc2. We also demonstrated that E-cadherin is required for the GSC centrosome orientation checkpoint, which prevents mitosis when centrosomes are not correctly oriented. We propose that E-cadherin orchestrates multiple aspects of stem cell behavior, including polarization of stem cells toward the stem cell-niche interface and adhesion of stem cells to the niche supporting cells.

## Introduction

Many stem cells are known to reside in a special microenvironment known as the niche to maintain their identity [1]. Stem cells often use adhesion molecules such as cadherins and integrins to stay anchored to the niche [2], [3]. In some model systems such as *Drosophila melanogaster* germline stem cells (GSCs), mitotic spindles are oriented toward the adherens junction formed between stem cells and the niche component [4]–[6]. This has led to speculation that the adherens junction might provide a polarity cue for spindle orientation. Such orientation leads to asymmetric stem cell division, with one daughter of the stem cell division staying within the niche and the other being displaced away from the niche.

In non-stem cell systems, abundant evidence shows that adherens junction components, including E-cadherin and β-catenin, are responsible for spindle orientation. For example, in epithelial cells of *Drosophila* embryos, spindle poles are closely associated with the adherens junctions present between neighboring cells, leading to orientation of spindles parallel to the epithelial surface and ensuring symmetric cell division [7]. Similarly, in a cultured epithelial model, the adherens junction orients mitotic spindles parallel to the epithelial layer [8]. In *Drosophila* sensory organ precursor cells, a series of asymmetric cell divisions leads to generation of different cell types, with E-cadherin functioning to orient mitotic spindles in the desired manner [9]. Recently, the E-cadherin/adherens junction was shown to be sufficient to polarize cells [10], [11], though centrosomes were oriented away from the adherens junctions in these cases.

Accumulating evidence suggests that adhesion molecules participate in spindle orientation in some stem cell models [3], including mammalian neuronal stem cells [12] and skin stem cells [13], both of which require integrins for correct spindle orientation. In *Drosophila* neuroblasts, spindle orientation correlates with contact with epithelial cells, implying that the adherens junction is involved in spindle orientation [14]. In addition, E-cadherin is concentrated at the interface between the neuroblast and ganglion mother cells (neuroblast daughters) [15]. However, addressing the functional significance of adhesion molecules in stem cell orientation has been challenging in many stem cell systems including *Drosophila* male GSCs, since these molecules are essential for the maintenance of stem cells within the niche. That is, stem cells are often lost and/or tissues are disorganized in the absence of adhesion molecules, hampering the assessment of their functions in stem cell polarity.

*Drosophila* male GSCs serve as an ideal model system for studying stem cell-niche interactions[16]. GSCs divide asymmetrically by orienting their mitotic spindles perpendicular to the adherens junction present between GSCs and the hub, a major niche component [4]. In male GSCs, the centrosomes are stereotypically oriented toward the adherens junction between the GSCs and hub cells, preparing for spindle orientation perpendicular to the hub cells. We have shown that correct centrosome orientation in male GSCs requires Apc2, a Drosophila homolog of adenomatous polyposis coli. Since Apc2 is thought to interact with both microtubules and the adherens junction component β-catenin, we proposed that Apc2 is a cortical anchor for the GSC centrosome at the hub-GSC junction and that the adherens junction provides a platform for Apc2 localization[4]. According to this hypothesis, the adherens junction not only anchors stem cells within the niche, but also provides a polarity cue for achieving asymmetric stem cell division. However, the requirement of E-cadherin in GSC polarity has not been tested since the absence of functional E-cadherin results in rapid loss of GSCs from the niche [17], hindering analysis of GSC polarity within the niche. Here we analyzed the role of E-cadherin in the polarization of *Drosophila* male GSCs using dominant-negative forms of E-cadherin, which disrupt stem cell polarity without perturbing cell-cell adhesion.

## Results

### Expression of dominant-negative E-cadherin does not perturb tissue architecture

**Figure 1:**
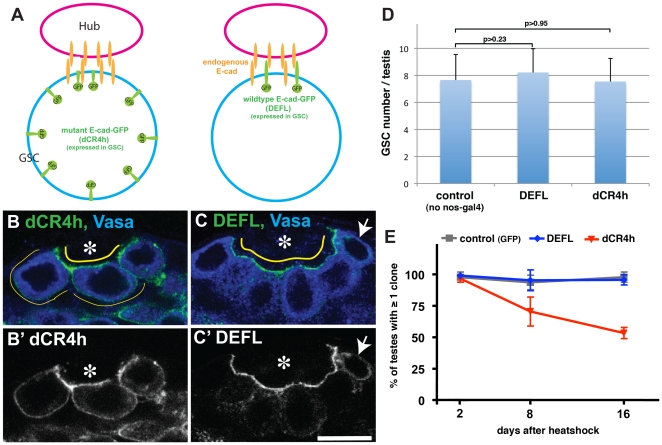
E-cad^dCR4h^ does not properly localize to the hub-GSC interface. (A) Experimental scheme. Wild type E-cad^DEFL^ or dominant-negative E-cad^dCR4h^ was expressed in GSCs using the germline-specific driver, nos-gal4. (B) E-cad^dCR4h^ was distributed throughout the entire GSC cortex, with a preference for the hub-GSC interface. The color of the text corresponds to the pseudocolored antibody staining or GFP signal in this and subsequent figures. GFP is shown in a separate panel (B') in gray scale. Vasa (germ cells). Asterisk (Hub). The scale bar represents 10 µm in this and subsequent figures. (C) E-cad^DEFL^ localizes to the hub-GSC interface. Arrow indicates GSCs with a higher expression level of E-cad^DEFL^. The localization of E-cad^dCR4h^ and E-cad^DEFL^ was monitored by GFP, since both E-cad^dCR4h^ and E-cad^DEFL^ contain GFP-tag at their C-termini. (D) GSC number was not affected by expression of E-cad^dCR4h^ or E-cad^DEFL^. Data are reported as mean ± S.D. in this and in subsequent graphs. n>45 testes per data point. (E) Percent of testes containing at least one GFP-positive clone at 2, 8, and 16 days after heatshock. n>60 testes per data point.

To test the function of E-cadherin in GSC polarity, we took advantage of a dominant-negative form of E-cadherin-GFP (E-cad^dCR4h^) that retains the transmembrane and intracellular domains but lacks part of the extracellular domain so that homotypic interactions are abolished (Figure 1A) [18]. Gal4/UAS-based expression of this molecule was reported to serve to perturb wild type DE-Cadherin function [19]. When E-cad^dCR4h^ was expressed using a germline-specific driver (nos-gal4 > UAS- E-cad^dCR4h^), it predominantly localized to the hub-GSC interface, though it also ectopically localized to the GSC cortex outside the hub-GSC interface (Figure 1B). In contrast, when wild type E-cadherin-GFP (E-cad^DEFL^) was expressed (nos-gal4 > E-cad^DEFL^ [18]), it localized exclusively to the hub-GSC interface (Figure 1C), as does endogenous E-cadherin [4]. In GSCs expressing higher levels of E-cad^DEFL^ (due to variability in nos-gal4-driven expression), an increased GFP signal was observed in the cytoplasm rather than in the entire GSC cortex (Figure 1C arrow and Supplementary Figure S1), suggesting that ectopic cortical localization of E-cad^dCR4h^ is not merely due to overexpression. Nevertheless, GSCs expressing E-cad^dCR4h^ remained attached to the hub cells, presumably because hub-GSC interactions were supported by endogenous E-cadherin (Figure 1A). GSC number was comparable between E-cad^dCR4h^-expressing testes and E-cad^DEFL^-expressing or control testes (without the nos-gal4 driver) (Figure 1D). However, when E-cad^dCR4h^ expression was induced in only a subset of GSCs, such clones tended to be lost more quickly than control GSCs (Figure 1E). This is possibly because E-cad^dCR4h^-expressing GSCs are at a disadvantage in competing with wild type GSCs [20]. Together, these data suggest that expression of E-cad^dCR4h^ compromises E-cadherin function (as evidenced by the loss of germline clones expressing E-cad^dCR4h^). Moreover, the finding that E-cad^dCR4h^ expression in all GSCs using the nos-gal4 driver did not disrupt tissue architecture or GSC attachment to the hub showed that E-cad^dCR4h^ could be used to test whether E-cadherin is required for GSC polarity.

### Expression of dominant-negative E-cadherin abolishes GSC centrosome orientation

**Figure 2:**
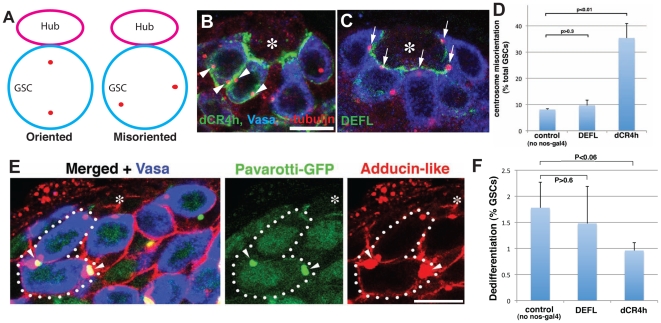
Expression of E-cad^dCR4h^ results in GSC centrosome misorientation. (A) Definition of GSC centrosome orientation. GSCs were scored as misoriented when neither of the two centrosomes was juxtaposed to the hub-GSC interface. (B) Examples of GSCs with misoriented centrosomes (arrowheads) upon expression of E-cad^dCR4h^. γ-tubulin (centrosomes). (C) Examples of GSCs with oriented centrosomes (arrows) upon expression of E-cad^DEFL^ (D) Quantification of GSC centrosome misorientation upon expression of E-cad^dCR4h^ or E-cad^DEFL^. Siblings without the nos-gal4 driver from the same cross served as controls. n>300 GSCs per data point. (E) An example of a dedifferentiating spermatogonium observed in testis expressing E-cad^dCR4h^. Multiple germs cells (including one attached to the hub) is connected by disintegrating ring canals and fusomes (arrowheads). Pavarotti-GFP marks ring canals, and Adducin-like marks fusome. (F) Quantification of dedifferentiation upon expression of E-cad^dCR4h^ or E-cad^DEFL^. Siblings without the nos-gal4 driver from the same cross served as controls. n>700 GSCs per data point.

Although E-cad^dCR4h^-expressing GSCs were well maintained, their centrosome was highly misoriented (∼35% misoriented centrosomes, Figure 2A right panel, B, D). This was striking contrast to wild type GSCs or E-cad^DEFL^-expressing GSCs that had stereotypically oriented centrosomes (Figure 2A, left panel [4], Figure 2C, D). This suggests that E-cadherin participates in GSC centrosome orientation. This result was confirmed using another dominant-negative form of E-cadherin, E-cad^dCR3h^ [18], which caused centrosome orientation defects similar to E-cad^dCR4h^ (Supplementary Figure S2). The expression of E-cad^dCR3h^ tended to be heterogeneous among GSCs, even within the same testis (Supplementary Figure S2). Specifically, when we scored centrosome orientation in GSCs with high and low (invisible) E-cad^dCR3h^ expression levels, we observed a strong correlation between the expression level and centrosome misorientation (Supplementary Figure S2). This suggests that the centrosome misorientation is a direct consequence of expressing dominant-negative E-cadherin. Due to the heterogeneous nature of E-cad^dCR3h^ expression, we decided to focus all subsequent analyses on E-cad^dCR4h^. We also decided not to examine loss-of-function E-cadherin clones, which are lost over time. In such clones, we would not be able to determine whether GSCs are still attached to the hub and thus, whether E-cadherin is directly required for stem cell polarity. We have previously reported that dedifferentiation increases centrosome misorientation [21]. As shown in Figure 2E and 2F, E-cad^dCR4h^ expression did not induce dedifferentiation of GSCs, assessed by the presence of GSCs connected with other germ cells with disintegrating fusomes and ring canals (Figure 2E). This result shows that centrosome misorientation is not attributable to dedifferentiation under these conditions. Taken together, these data demonstrate that expression of dominant-negative E-cadherin disrupts centrosome orientation in GSCs.

### Apc2 functions as a cortical anchor for the GSC Centrosome and is mislocalized upon expression of dominant-negative E-cadherin

**Figure 3:**
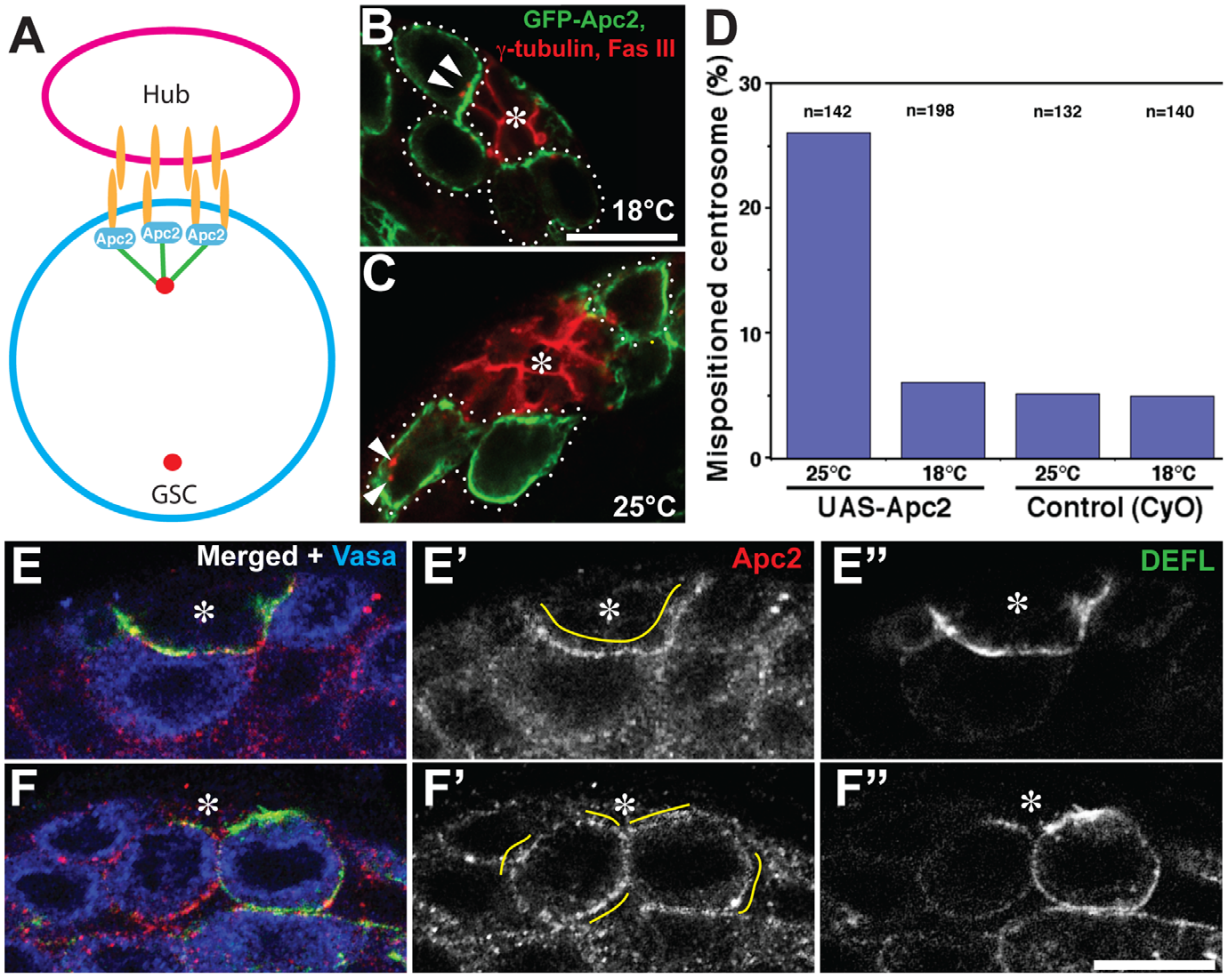
Expression of E-cad^dCR4h^ results in even cortical distribution of the Apc2 protein. (A) Model of Apc2 linking the adherens junction and the centrosome. Orange; cadherin. Green; microtubule. (B, C) GFP-Apc2 expressed by nos-gal4 at 18°C preferentially localized to the hub-GSC cortex junction (B), while it was evenly distributed throughout the GSC cortex when expressed at 25°C (C). Oriented centrosomes (at 18°C) and misoriented centrosomes (at 25°C) are indicated with arrowheads. GSCs in the right focal plane to judge its cortical localization are marked by dotted lines (4 GSCs in panel B, 3 GSCs in panel C). The hub is stained with Fas III. (D) Quantification of centrosome misorientation upon expression of GFP-Apc2 at 18°C or 25°C. Essentially the same results were obtained in more than three repeated experiments. (E) Apc2 localizes to the hub-GSC junction in testes expressing E-cad^DEFL^. (F) Apc2 is evenly distributed throughout the GSC cortex in testes expressing E-cad^dCR4h^. Cortical sites with prominent Apc2 localization are indicated with yellow lines.

Next, we investigated the molecular mechanisms by which E-cad^dCR4h^ disrupts GSC centrosome orientation. We have previously shown that Apc2, a homolog of Adenomatous polyposis coli [15], [22]–[25], is localized to the hub-GSC interface [4]. We hypothesized that Apc2 anchors the microtubules emanating from the centrosome (Figure 3A). When GFP-Apc2 was expressed in the germline (nos-gal4 > UAS-GFP-Apc2) at 18°C, it preferentially localized to the hub-GSC junction (Figure 3B). However, upon overexpression at 25°C, GFP-Apc2 was evenly distributed throughout the GSC cortex (Figure 3C). Strikingly, this resulted in a high frequency of GSC centrosome misorientation (Figure 3D). These data suggest that Apc2 functions as a cortical anchor for the centrosome.

Analysis of Apc2 localization in GSCs expressing E-cad^dCR4h^ revealed that Apc2 was evenly distributed throughout the cortex of these cells (Figure 3F). This contrasted to the preferential localization of Apc2 at the hub-GSC interface in E-cad^DEFL^-expressing GSCs (Figure 3E), a localization also seen in wild type GSCs. These data imply that E-cad^dCR4h^ recruits Apc2 to ectopic cortical sites, leading to centrosome misorientation.

### Expression of dominant-negative E-cadherin results in misoriented spindles

**Figure 4:**
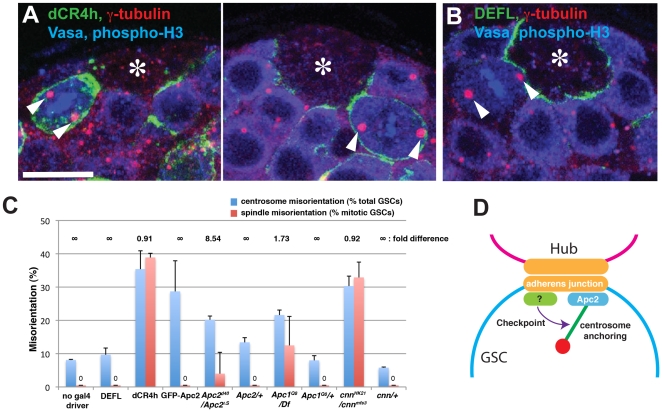
Expression of E-cad^dCR4h^ results in misoriented spindle in mitotic GSCs. (A) Examples of GSCs with misoriented spindles upon expression of E-cad^dCR4h^. Phospho-histone H3 (mitotic chromosomes). (B) An example of a GSC with an oriented spindle in an E-cad^DEFL^-expressing testis. (C) Quantification of GSC centrosome orientation (% misoriented centrosomes/total interphase GSCs) and spindle misorientation (% misoriented spindles/total mitotic GSCs). The fold difference (centrosome misorientation frequency/spindle misorientation frequency) is shown at the top of graph. n>250 GSCs per data point for centrosome orientation. n>30 mitotic GSCs per data point for spindle orientation. For *apc2* and *cnn* mutants, heterozygous siblings from the same cross served as controls. (D) Model for E-cadherin function in multiple stem cell behaviors. The E-cadherin-based adherens junction serves as a polarity cue for stem cell orientation and also maintains stem cells in the niche. The intracellular domain of E-cadherin recruits Apc2 (directly or indirectly) and factor(s) that participate in the centrosome orientation checkpoint.

While scoring centrosome orientation in GSCs expressing E-cad^dCR4h^, we noticed that these GSCs had a high frequency of misoriented spindles during mitosis (∼39%, Figure 4A, C), while GSCs expressing E-cad^DEFL^ did not (0%, Figure 4B, C). We have recently shown that GSCs with misoriented centrosomes do not enter mitosis until the orientation is corrected, pointing to the presence of a novel checkpoint that monitors centrosome orientation (“the centrosome orientation checkpoint”) [21]. However, E-cad^dCR4h^ –expressing GSCs had similar level of GSC centrosome misorientation (∼35%) and spindle misorientation (∼39%), suggesting that these GSCs do not delay mitosis even when centrosomes are misoriented. Together, these results suggest that E-cad^dCR4h^ overrides this checkpoint.

The high frequency of misoriented spindles resulting from expression of E-cad^dCR4h^ cannot be explained by ectopic localization of Apc2, since the overexpression of Apc2 did not lead to spindle misorientation (Figure 4C). Consistent with the idea that Apc2 does not participate in the centrosome orientation checkpoint, *apc2* mutants also maintained significant checkpoint activity. Although *apc2^d40/^apc2^N175K^* mutants have a high centrosome misorientation frequency (∼20%) as reported previously [4], the spindle misorientation frequency is lower (4%) than centrosome misorientation frequency (Figure 4C). This indicates that *apc2* mutants are capable of delaying cell cycle progression following centrosome misorientation. It should be noted that the frequency of spindle misorientation in *apc2^d40/^apc2^N175K^* mutants was somewhat lower than previously reported (∼10%), likely due to a change in fixation procedure (see Materials and Methods). Regardless, the large fold difference (centrosome misorientation/spindle misorientation) under previous and current experimental conditions demonstrates that the *apc2* mutant is capable of sensing centrosome misorientation and subsequently delaying mitosis. This is a stark contrast to GSCs expressing E-cad^dCR4h^, which have comparable spindle and centrosome misorientation frequencies (Figure 4C).

To gain further insight into the molecular requirements for this checkpoint, we investigated other mutants known to be defective in centrosome orientation. We previously reported that, like the *apc2* mutant, *cnn* and *apc1* mutants exhibit centrosome and spindle misorientation [4]. Cnn is an integral component of pericentriolar material [26]–[28]. The *cnn^HK21/mfs3^* mutant had a high frequency of centrosome misorientation (30.3%) and a similar frequency of spindle misorientation (32.9%) (Figure 4C). This indicates that *cnn* mutants do not properly delay cell cycle progression upon centrosome misorientation. Apc1 protein has been shown to localize to the spindle pole in mitotic GSCs [4]. We found that the *apc1* mutant showed only a 1.7-fold difference in centrosome and spindle misorientation (Figure 4C), indicating that the *apc1* mutant is partially defective in the centrosome orientation checkpoint. Together, these results show that misoriented centrosomes do not necessarily lead to misoriented spindles, due to the presence of the centrosome orientation checkpoint. They also show that E-cadherin and Cnn (and possibly Apc1) are essential components of this checkpoint, while Apc2 is not.

## Discussion

In this study, we demonstrated that E-cadherin is important for polarization of GSCs within the niche, a function that has been masked by its requirement in GSC maintenance. We showed that expression of a dominant-negative form of E-cadherin (E-cad^dCR4h^ and E-cad^dCR3h^) that is incapable of homotypic interactions due to a truncated extracellular domain results in a high frequency of centrosome misorientation. The finding that Apc2 protein was delocalized in these animals may at least partly explain the centrosome misorientation phenotype.

We also showed that expression of E-cad^dCR4h^ leads to a high frequency of spindle misorientation. This suggests that E-cadherin participates in the mechanism that delays mitosis when centrosomes are misoriented (the centrosome orientation checkpoint). Alternatively, the checkpoint might monitor the interaction between E-cadherin and the centrosome. Consequently, when dominant-negative E-cadherin (E-cad^dCR4h^) anchors the centrosomes to ectopic cortical sites, the checkpoint might “misunderstand” that the centrosomes are correctly oriented, leading to mitosis with misoriented spindles. Apc2 apparently does not play a significant role in this process, as evidenced by the fact that spindle orientation was normal (or close to normal) in *apc2* mutants or Apc2-overexpressing GSCs, both of which exhibit a high frequency of centrosome misorientation. This suggests that E-cadherin participates in the checkpoint through factor(s) other than Apc2 (Figure 4D). It is worth noting that the ratio of E-cad^dCR4h^ at the hub-GSC interface versus the lateral membrane (Figure 1B) is similar to that of GFP-Apc2 protein expressed at 18°C (Figure 3B); however, expression of E-cad^dCR4h^ leads to centrosome misorientation, while expression of GFP-Apc2 (at 18°C) does not. Thus, E-cad^dCR4h^ is apparently more potent in misorienting the centrosomes. These findings are consistent with the notion that E-cadherin anchors Apc2 in parallel with other factor(s) that function in anchoring the centrosome and regulate the checkpoint. Future studies are needed to identify such factor(s).

In our previous study, we have reported that *cnn* mutant GSCs show high frequency of misoriented spindles, leading to symmetric GSC divisions and thus an increase in GSC number [4]. However, we did not observe an increase in GSC number upon expression of E-cad^dCR4h^, in spite of high frequency of spindle misorientation. We speculate that E-cad^dCR4h^-expressing GSCs may need a larger cortical area to stay adhered to the hub cells due to its disadvantage in adhesion. In *cnn* mutant, extra GSCs were observed to be “over-crowded” around the hub cells, attaching to the hub with a small cortical area, since the hub size does not increase.

The current study also revealed that the two centrosomal proteins, Cnn and Apc1, differentially contribute to centrosome orientation and the centrosome orientation checkpoint. While the checkpoint was completely abolished in the *cnn* mutant, the *apc1* mutant appeared to retain some level of checkpoint activity. The selective requirement for certain molecules in centrosome orientation and the centrosome orientation checkpoint implies that the mechanism by which the centrosome is anchored to the adherens junction is separable from the mechanism responsible for sensing centrosome orientation. The involvement of E-cadherin in the checkpoint might suggest that the sensing function is located at the adherens junctions, whereas the involvement of Cnn and Apc1 suggests that the checkpoint activity might be located at the centrosome/spindle pole. Alternatively, as is the case for the kinetochore checkpoint [29], it might be the tension of the microtubules linking the adherens junction and centrosome that is monitored by the checkpoint. We propose that the adherens junction formed between the niche component (hub cells) and stem cells (GSCs) serves as a platform for multiple functions essential for stem cells: 1) maintenance of stem cells within the niche by physically anchoring the stem cells, 2) polarization of stem cells with respect to the niche, and 3) localization of factor(s) required for monitoring stem cell polarity.

## Materials and Methods

### Fly husbandry and strains

All fly stocks were raised on standard Bloomington medium at 25°C. The following fly stocks were used: UAS-DEFL, UAS-dCR4h, UAS-dCR3h (a gift from H. Oda)[18], UAS-GFP-Apc2/TM3 (a gift from M. Bienz), *cnn^HK21^*/CyO, *cnn^mfs3^*/CyO [26]-[28], *apc2^d40^*/TM3, *apc2^N175K^*/TM6b [22]–[24], nos-gal4 [30] (obtained from the Bloomington Stock Center) and hs-FLP; UAS-GFP Act-FRT-stop-FRT-gal4 (a gift from Y. Cai).

### Immunofluorescence microscopy

Samples were fixed for 30–60 min with 4% formaldehyde in PBS and permeabilized for 30 min in PBST (0.1% Triton X-100 in PBS). Samples were then incubated overnight at 4°C with primary antibodies, washed with PBST (3 times, 20 min), incubated overnight at 4°C with AlexaFluor-conjugated secondary antibodies (1∶200, Molecular Probes), and washed again with PBST (3 times, 20 min). Samples were then mounted in Vectashield (H-1200, Vector Laboratory). The primary antibodies used were mouse anti-γ-tubulin (1∶100; GTU-88, Sigma), mouse anti-Fasciclin III [1∶20, developed by C. Goodman and obtained from the Developmental Studies Hybridoma Bank (DSHB)], mouse anti-Adducin-like (1∶20, Developed by H. D. Lipshitz and obtained from DSHB), rabbit anti-Thr3-phosphorylated Histone H3 (1∶200, Upstate), goat anti-Vasa (1∶100; dC-13, Santa Cruz Biotechnology), and rabbit anti-Vasa (1∶100, Santa Cruz Biotechnology). Images were captured using a Leica TCS SP5 confocal microscope with a 63x oil immersion objective (NA = 1.4) and processed using Adobe Photoshop.

As mentioned in the Results, a change in fixation procedure decreased spindle misorientation (but not centrosome orientation) in *apc2, apc1*, and *cnn* mutants. In the original fixation procedure, testes were fixed after being squashed between the coverslip and slide [4]. However, in the current study, samples were fixed as whole-mount tissue, without any structural perturbation taking place. The old method (in which a pressure was applied to the tissue before fixation) did not affect spindle orientation in wild type testes but did affect spindle orientation in mutant backgrounds, which presumably had somewhat compromised spindle attachment.

### Clonal analysis

We subjected hs-FLP; UAS-GFP Act-FRT-stop-FRT-gal4/UAS-E-cad^dCR4h^ or UAS-E-cad^DEFL^ to heatshock at 37°C for 120 min. The number of testes containing any GFP-positive clone was then determined. Under these experimental conditions, multiple clones were often induced in each testis.

## Supporting Information

Figure S1 Wild type cadherin (DEFL) localizes to the hub-GSC interface, even when overexpressed. An example of a GSC overexpressing E-cadDEFL (arrow). Excess E-cadDEFL was observed in the cytoplasm rather than at the GSC cortex.
(0.18 MB PDF)

Figure S2 Expression level of E-caddCR3h correlates with centrosome misorientation. A) An example of testis apical tip, with heterogenous expression of E-caddCR3h. Arrows indicate GSCs with E-caddCR3h visible at the lateral cortex, arrowheads indicate GSCs with E-caddCR3h only at hub-GSC interface (both scored as dCR3hhigh), and open arrows indicate GSCs with no visible E-caddCR3h at all (scored as dCR3hlow). B) Higher expression of E-caddCR3h correlated with high frequency of centrosome misorientation.
(2.19 MB PDF)
