# Supplementary figures and images for: E-Cadherin Is Required for Centrosome and Spindle Orientation in Drosophila Male Germline Stem Cells
Source: PLoS One. 2010 Aug 31;5(8):e12473. doi: 10.1371/journal.pone.0012473 (PMC2930853; doi:10.1371/journal.pone.0012473)

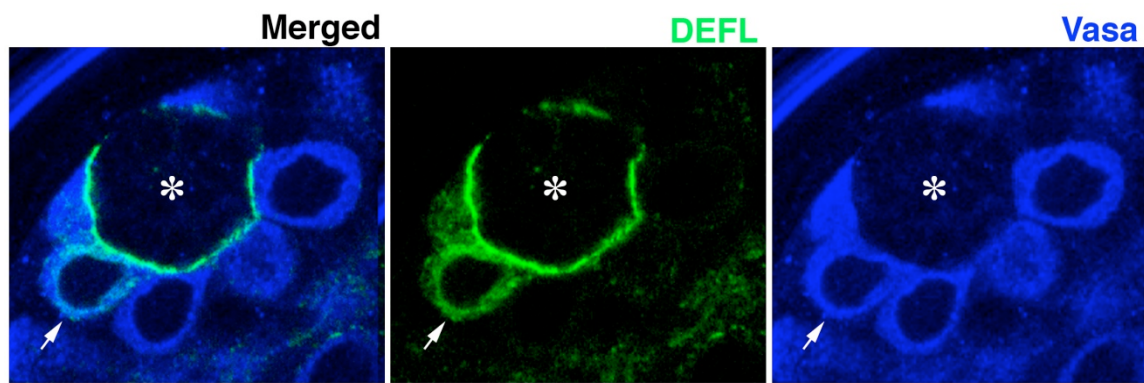

Supplement: Figure S1 — Wild type cadherin (DEFL) localizes to the hub-GSC interface, even when overexpressed. An example of a GSC overexpressing E-cadDEFL (arrow). Excess E-cadDEFL was observed in the cytoplasm rather than at the GSC cortex. (0.18 MB PDF) [file pone.0012473.s001.pdf]

**A** Merged with *Vasa*, *Fas III*,  $\gamma$ -tubulin

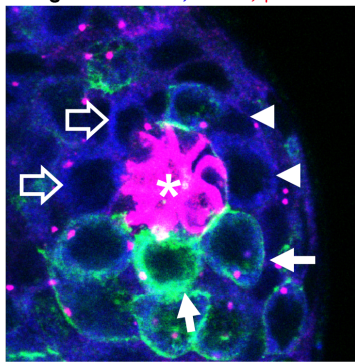

*dCR3h*

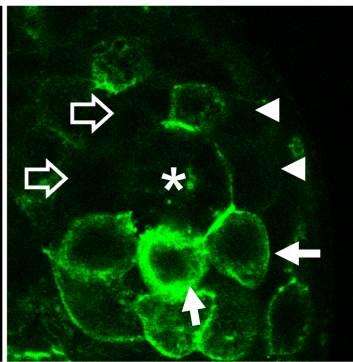

**B**

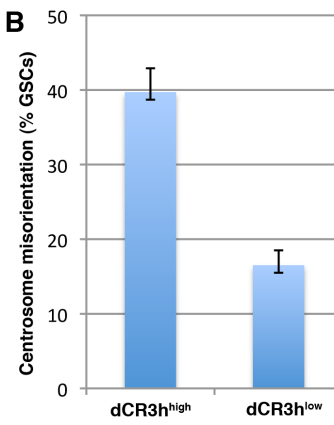

Supplement: Figure S2 — Expression level of E-caddCR3h correlates with centrosome misorientation. A) An example of testis apical tip, with heterogenous expression of E-caddCR3h. Arrows indicate GSCs with E-caddCR3h visible at the lateral cortex, arrowheads indicate GSCs with E-caddCR3h only at hub-GSC interface (both scored as dCR3hhigh), and open arrows indicate GSCs with no visible E-caddCR3h at all (scored as dCR3hlow). B) Higher expression of E-caddCR3h correlated with high frequency of centrosome misorientation. (2.19 MB PDF) [file pone.0012473.s002.pdf]
